# Trp-Containing Antibacterial Peptides Impair Quorum Sensing and Biofilm Development in Multidrug-Resistant *Pseudomonas aeruginosa* and Exhibit Synergistic Effects With Antibiotics

**DOI:** 10.3389/fmicb.2021.611009

**Published:** 2021-02-11

**Authors:** Dejing Shang, Xue Han, Wanying Du, Zhiru Kou, Fengquan Jiang

**Affiliations:** ^1^School of Life Sciences, Liaoning Normal University, Dalian, China; ^2^Liaoning Provincial Key Laboratory of Biotechnology and Drug Discovery, Liaoning Normal University, Dalian, China; ^3^Clinical Laboratory Department of the First Affiliated Hospital, Dalian Medical University, Dalian, China

**Keywords:** multidrug-resistant *Pseudomonas aeruginosa*, Trp-containing peptide, quorum sensing, virulence factor, biofilm, synergy

## Abstract

*Pseudomonas aeruginosa* uses quorum sensing (QS) to control virulence, biofilm formation and antibiotic efflux pump expression. The development of effective small molecules targeting the QS system and biofilm formation represents a novel attractive strategy. In this present study, the effects of a series of Trp-containing peptides on the QS-regulated virulence and biofilm development of multidrug-resistant *P. aeruginosa*, as well as their synergistic antibacterial activity with three classes of traditional chemical antibiotics were investigated. The results showed that Trp-containing peptides at low concentrations reduced the production of QS-regulated virulence factors by downregulating the gene expression of both the las and rhl systems in the strain MRPA0108. Biofilm formation was inhibited in a concentration-dependent manner, which was associated with extracellular polysaccharide production inhibition by downregulating *pelA*, *algD*, and *pslA* transcription. These changes correlated with alterations in the extracellular production of pseudomonal virulence factors and swarming motility. In addition, the combination of Trp-containing peptides at low concentration with the antibiotics ceftazidime and piperacillin provided synergistic effects. Notably, L11W and L12W showed the highest synergy with ceftazidime and piperacillin. A mechanistic study demonstrated that the Trp-containing peptides, especially L12W, significantly decreased β-lactamase activity and expression of efflux pump genes *OprM*, *MexX*, and *MexA*, resulting in a reduction in antibiotic efflux from MRPA0108 cells and thus increasing the antibacterial activity of these antibiotics against MRPA0108.

## Introduction

The gram-negative bacterium *Pseudomonas aeruginosa* is an opportunistic human pathogen found in hospital-acquired infections, such as cystic fibrosis (CF) and burn and wound infections, particularly in immunocompromised patients (Lyczak et al., 2000; Breidenstein et al., 2011). With the indiscriminate use of antibiotics to treat *P. aeruginosa*-caused infections, there is a widespread appearance of the multidrug resistant (MDR) strains, leading to a significantly increased incidence of antibiotic resistance. In addition to innate resistance mechanisms and acquired resistance to antibiotics (Strateva and Yordanov, 2009), *P. aeruginosa* contributes antibiotic resistance through its ubiquitous ability to thrive in different environmental conditions by virtue of its highly complex regulatory networks and ability to form communities called biofilms. The quorum sensing (QS) regulates the population density of bacteria by a highly organized cell-to-cell signaling communication system in which constitutively expressed QS signaling molecules regulate virulence factors production to respond to changes in *P. aeruginosa* population density (Lee and Zhang, 2015; Pattnaik et al., 2018). When the bacterial population densities are low, virulence gene expression is not activated to avoid pathogen detection and immune stimulation against virulence factors. Once a threshold level of signal molecules has been reached, coordinated gene expression is induced. This system gives bacteria ample time for colonization and establishment in the host. Such virulence factors can affect biofilm formation and swarming motility by the extracellular polymeric substance (EPS), oxygen availability and matrix density (Bjarnsholt et al., 2010; Coenye, 2010; Brackman and Coenye, 2015).

*P. aeruginosa* QS depends on 3 well-defined and highly characterized signaling systems, i.e., the *lasI*/*lasR*, *rhlI*/*rhlR*, and *pqs*/*mvfR* systems, for the communal response to extracellular signaling autoinducer molecules, such as 3OC12-homoserine lactone (C12-HSL) and N-butyryl-L-homoserine lactone (C4-HSL) (Pattnaik et al., 2018). In the *las* system, the transcription factor *lasR* is first activates by the autoinducer C12-HSL, and then *lasR* drives *lasI* expression and triggers the production of exotoxin A, the LasA protease and the LasB elastase. Similarly, in the *rhl* system, the autoinducer C4-HSL increases *rhlI* expression by the binding and interaction of C4-HSL with *rhlR* and induces controlled production of the LasB elastase, rhamnolipids, pyocyanin, and cytotoxic lectins that play crucial roles in virulence and biofilm formation and development. The *rhl* system is also regulated by the *lasR*/C12-HSL complex at the transcriptional and posttranscriptional levels. As present, QS has not been found in humans, so it will be a specific antibacterial target. Therefore, it seems to be highly appropriate and significant to develop novel antimicrobial drugs for use as potent antiinfectives targeting QS systems and associated virulence factors.

Antimicrobial peptides (AMPs) are composed of short, positively charged amino acid residues, which represent one of the most promising alternative agents for conventional antibiotics because they quickly kill a broad spectrum of microorganisms, including species resistant to other antimicrobials (Fjell et al., 2011; Abd-El-Aziz et al., 2015). Although currently available AMPs exhibit limitations, such as protease instability, high production costs and inactivity under physiological conditions, it is important to develop more effective AMPs as a novel class of antibiotics (Pasupuleti et al., 2011). Many AMPs, especially cationic AMPs with an amphipathic arrangement of cationic and hydrophobic residues, can directly cover bacterial membranes, alter membrane permeability, and create pores across the membrane, leading to cell content leakage and bacterial death (Yeaman and Yount, 2003). However, a previous study reported that colistin, a cationic AMP, altered the expression of 30 genes associated with virulence and bacterial colonization in chronic *P. aeruginosa* infection (Cummins et al., 2009). Clearly, this finding points to a non-membrane disruption mechanism of AMPs in combating MDR bacteria.

Tryptophan (Trp) residues are often found in naturally occurring AMPs, and their aromatic side chain can form hydrogen bonds with a dipole moment of ∼21 D in an interfacial membrane region (Khandelia and Kaznessis, 2007). This special feature makes Trp-containing peptides partition into the bilayer interface, and Trp-containing peptides been proven effective as antibacterial agents (Chan et al., 2006; Rydberg et al., 2012). A series of Trp-containing peptides has been designed and synthesized by replacing isoleucine residues or leucine residues with Trp residues at different positions of L-K6, a AMP derived from the frog skin peptide temporin-1CEb in our lab (Shang et al., 2012; Bi et al., 2013, 2014). I1W and I4W exhibited stronger antibacterial activity against both gram-positive and gram-negative bacteria than the other peptides, but L11W and L12W had only moderate or low antibacterial activity. I1WL5W and I4WL5W had a broad-spectrum antibacterial activity. We have also determined that the position of Trp residues rather than the number of Trp residues in these peptides play a key role in the antibacterial activity (Bi et al., 2013, 2014). The present study focused on the effects of Trp-containing AMPs on virulence factors and biofilm formation and development regulated by QS in MDR *P. aeruginosa* (MRPA0108) and aimed to provide an improved prospect for developing more effective antiinfective drugs targeting bacterial virulence.

## Materials and Methods

### Materials

Peptides were purchased from GL Biochemistry Inc. (Shanghai, China). The purity of peptides was ≥5%. Amino acid sequences and physical characteristics for peptides are shown in Supplementary Table S1. TRIzol reagent and Super Script^TM^ III kits were purchased from TaKaRa Inc. (Dalian, China). Ceftazidime, piperacillin and levofloxacin were purchased from Solarbio Inc. (Beijing, China). C4-HSL standards is purchased from Sigma (Sigma, China). All other reagents were of analytical grade. The MDR *P. aeruginosa* (MRPA) strain 0108 (MRPA0108) was obtained from the Clinical Laboratory Department, the First Affiliated Hospital of Dalian Medical University. MRPA0108 exhibits resistance to gentamicin, kanamycin, ampicillin, cefepime, streptomycin, tetracycline, amoxicillin, and ciprofloxacin. Identification and antimicrobial susceptibility testing (AST) of MRPA0108 were performed by an automated MicroScan WalkAway 96 Plus system (Siemens Ltd., Germany) at the First Affiliated Hospital of Dalian Medical University.

### Real-Time Quantitative PCR Analysis

Mid-logarithmic (mid-log) growth-phase MRPA0108 cells (1 × 10^8^ CFU/ml) were incubated with the different concentrations of peptide at 37°C for 18 h. The bacterial cells were collected by centrifugation at 13,000×*g* and digested in lysis buffer containing 10 mg/ml lysozyme. Total RNA was isolated by using a TRIzol^®^ Max^TM^ Kit (Invitrogen, Shanghai, China) according to the manufacturer’s instructions and subsequently reverse transcribed into cDNA using a Prime Script RT Master Mix Kit (Takara, Dalian, China). Real-time PCR was performed in an ABI QuantStudio^TM^ 5 sequence detection system (Applied Biosystems, United States) using 2 × SYBR Premix Ex Taq II (Solarbio Science and Technology Co., Ltd.). The PCR cycling conditions were as follows: 95°C for 30 s and 40 cycles of 5 s at 95°C, 30 s at 60°C, and 20 s at 72°C. The fold changes calculated according to the comparative cycle threshold (Ct) method were used for quantifying gene expression, normalizing to the expression of the housekeeping gene *rpsL*. PCR primers, including those for *lasA*, *lasB*, *rhlA*, *rhlB*, *pelA*, *algD*, *psl*, *oprD*, *oprM, mex*A, and *mexB*, were synthesized by Sangon Biotech (Shanghai, China) and are presented in Supplementary Table S2.

### Determination of QS-Regulated Virulence Factor Production

Mid-log growth-phase MRPA0108 cells (1 × 10^5^ CFU/ml) were incubated in the presence or absence of peptide at 1/4 × the minimal inhibitory concentration (MIC) at 37°C in a shaking culture for 24 h. The culture supernatant was obtained after centrifugation at 12,000×*g* for 10 min. (1) LasB elastase activity was measured using elastin-Congo red (ECR) as the substrate, as described by Pattnaik et al. (2018). Briefly, 100 μl of the culture supernatant was added to 900 μl of ECR buffer (100 mM Tris-HCl, 1 mM CaCl_2_, pH 7.5) containing 10 mg of ECR and then incubated with shaking at 37°C for 18 h. Then, 100 μl of 0.12 M EDTA was added to stop the reaction. The insoluble ECR was removed by centrifugation, and the supernatant absorbance was measured at 495 nm. The optical density (OD) 495 nm/OD 600 nm represents the elastase activity. (2) LasA protease activity was measured as follows (Pattnaik et al., 2018): first, the culture supernatant was filtered to remove the bacterial cells, and then 150 μl of sterile-filtered supernatant was added to 2% azocasein (prepared in 50 mM Tris-HCl) and incubated for 4 h at 37°C. Trichloroacetic acid (TCA, 10%) was added to precipitate the undigested substrate. After centrifugation at 10,000 rpm for 10 min, 1 M NaOH was added to the supernatant, and the relative protease activity was measured at OD 440 nm. (3) The pyocyanin assay was based on the absorbance of excreted pyocyanin at 520 nm in an acidic solution (Kong et al., 2005). Three milliliters of culture supernatant were mixed with 2 ml of chloroform for 20 min, and then the pyocyanin from the chloroform phase was reextracted using 0.2 M HCl for 15 min to obtain a pink-colored pyocyanin solution. The OD 520 nm was measured to evaluate the change in pyocyanin content. (4) Rhamnolipid detection was carried out as previously described (Wittgens et al., 2011). Briefly, 500 μl of culture supernatant was mixed with 500 μl of ethyl acetate and vortexed vigorously for 15 s to extract rhamnolipid. After centrifugation at 10,000×*g* for 5 min at 4°C, the organic phase containing rhamnolipids was obtained and evaporated at room temperature overnight. The rhamnolipid extract dissolved in ultrapure water was mixed with orcinol reagent (0.19% orcinol in 53% H_2_SO_4_) and incubated at 80°C for 30 min. The absorbance was measured at 420 nm. (5) The level of C4-HSL was quantified by GC-MS as previously described (Guo et al., 2012).

### Swarming Motility Assay

The swarming motility of MRPA0108 was assessed as described by O’May and Tufenkji (2011), with slight modifications. Briefly, swarming agar medium (1% tryptone, 0.5% NaCl, and 0.5% agar) was prepared in the presence or absence of peptide at a final concentration of 1/4 × MIC. MRPA0108 cells from an overnight culture grown on LB agar plates were placed directly on the agar surface (center) with a sterile toothpick so that the motility within the semisolid agar could be evaluated. After 24 h of incubation at 37°C, the diameters of swarming motility were observed and measured. LB liquid medium and erythromycin were used as negative control and positive controls, respectively. All experiments were repeated three times.

### Observation of Bacterial Biofilms Using Confocal Laser Microscopy

Biofilms of MRPA0108 were visualized by confocal microscopy. Mid-log growth-phase bacteria were diluted to 1 × 10^7^ CFU/ml. One milliliter of diluted culture and peptide (at a final concentration of 1 × MIC or 2 × MIC) were incubated in 12-well plates with chambered coverglass at 37°C for 24 h. LB liquid medium and erythromycin were used as negative and positive controls, respectively. After incubation, the supernatant was removed, and the slides were washed and stained in a dark environment for 30 min using a LIVE/DEAD BacLight^TM^ Bacterial Viability Kit (Invitrogen, Life Technologies, Shanghai, China). The slides were washed three times and imaged with a confocal laser scanning microscope (LSM 710, Carl Zeiss, Germany) with excitation/emission wavelengths of 480/500 and 490/635 nm for SYTO9 and propidium iodide (PI), respectively. SYTO9 was used to indicate live bacteria, and PI was used to mark dead bacteria.

### Biofilm Formation Assay

The biofilm formation of the MRPA0108 strain was assessed as described previously (Shang et al., 2017). Briefly, 50 μl of a mid-log growth-phase bacterial solution (1 × 10^5^ CFU/ml) was placed into a 96-well microtiter plate and incubated overnight with 50 μl of peptide at a final concentration of 1/8 × MIC, 1/4 × MIC, 1/2 × MIC, or 1 × MIC. LB liquid medium and erythromycin were used as negative and positive controls, respectively. After 24 h of incubation at 37°C, planktonic bacteria were carefully removed, and the biofilms were fixed with formaldehyde for 15 min and stained with 0.1% (w/v) crystal violet (CV) dye for 5 min. Before being quantified by reading the absorbance at 590 nm in a microtiter plate reader (Varioskan Flash Microplate Reader, Thermo Fisher Scientific Co., Beijing), biofilms were washed and solubilized with 95% ethanol. The OD 590 nm was determined as a measure of biofilm biomass.

Degradation of 1-day-old biofilms was measured as described previously (Breij et al., 2018). First, biofilms were grown by a 24 h incubation of mid-log growth-phase culture (1 × 10^5^ CFU/ml) at 37°C. Next, planktonic bacteria were removed by washing with PBS. The biofilms were incubated with peptides at a final concentration of 1/8 × MIC, 1/4 × MIC, 1/2 × MIC, or 1 × MIC at 37°C for 24 h and then fixed, stained and quantified as described above. All experiments were repeated three times. LB liquid medium and erythromycin were used as negative control and positive controls, respectively.

### Determination of the Polysaccharides in Biofilms

First, mid-log growth-phase bacteria (1 × 10^5^ CFU/ml) were incubated with peptide at a final concentration of 1/8 × MIC, 1/4 × MIC, 1/2 × MIC, or 1 × MIC at 37°C overnight. Exopolysaccharides were determined using the phenol-H_2_SO_4_ method. Briefly, after incubation, the cultures were centrifuged at 10,000 rpm for 15 min, and the pellets were resuspended in high-salt buffer (10 mM KPO4, 5 mM NaCl, 2.5 mM MgSO4, pH 7.0), followed by recentrifugation at 10,000 rpm for 30 min. The resulting supernatant was mixed with three volumes of chilled ethanol (100%) and incubated overnight at 4°C. The precipitated exopolysaccharide was added to a mixture containing cold phenol and H_2_SO_4_, and the absorbance of the solution was measured at 490 nm. Alginate content was determined using the H_2_SO_4_-carbazole method. Briefly, after incubation, the precooled cultures were mixed with an H_2_SO_4_ solution (4:1) and vortexed in ice water, and then 0.2% carbazole dissolved in ethanol was added. The mixture was revortexed and incubated at 55°C for 30 min, and the OD 530 nm was measured.

Psl content was detected using the liquid Congo red (CR, Sigma-Aldrich, Shanghai, China) method described by Ma et al. (2006). Briefly, mid-log growth-phase bacteria (1 × 10^5^ CFU/ml) were incubated in no-salt LB liquid medium containing 40 μg/ml CR in the presence or absence of peptide at a final concentration of 1/8 × MIC, 1/4 × MIC, 1/2 × MIC, or 1 × MIC at 37°C overnight. After the absorbance was measured at 600 nm, the cultures were centrifuged at 14,000 rpm for 5 min, and then the absorbance of the supernatant was measured at 490 nm to determine the binding of CR and bacterial cells.

### Checkerboard Assay

The synergistic effect of peptides and antibiotics was evaluated using the checkerboard method, in which the fractional inhibitory concentration index (FICI) was used to analyze the type of interaction between compounds (Berditsch et al., 2015). A 100 μl inoculum of 10^5^ CFU/ml planktonic bacteria was added to the respective wells of 96-well microtiter plates, and then serial dilutions of peptide and antibiotic at 100 μl volumes were added to each well and incubated at 37°C for 24 h. The OD 600 nm value was measured with a microplate plate reader (Varioskan Flash Microplate Reader, Thermo Fisher Scientific Co., Beijing) as an indicator of growth inhibition. The resulting checkerboard contained each combination of peptide and antibiotic in 9 doubly increasing concentrations, with wells containing the highest concentration of each antibiotic at opposite corners. The concentrations tested for every combination of peptide-antibiotic were their respective MICs. MH medium and the peptide/antibiotic combination served as negative and positive controls, respectively. The FICI is calculated as FIC_*a*_ plus FIC_*b*_, where FIC_*a*_ is the MIC of the peptide in the presence of antibiotics/the MIC of the peptide alone, and FIC_*b*_ is the MIC of the antibiotic in the presence of peptide/the MIC of the antibiotic alone. Drug interactions were defined as follows: synergy, FICI ≤ 0.5; additive effect, 0.5 < FICI ≤ 1; and indifference, 1 < FICI ≤ 2 (Hollander et al., 1998; Yoon et al., 2004). The results were recorded in at least three independent experiments, and the median FICI values were used in the analysis.

### Intracellular Antibiotic Assay by HPLC

Mid-log growth-phase bacteria were diluted to 1 × 10^8^ CFU/ml and treated with I1W or L12W at concentrations ranging from 3.13 to 50 μM for 12 h at 37°C. After induction, the cultures were harvested by centrifugation at 12,000 rpm for 2 min, washed three times with PBS buffer (pH 7.2), and then incubated with lysozyme (20 mg/ml) at 37°C for 1 h in a water bath. After centrifugation at 12,000 rpm for 5 min, the supernatant of the cell lysates was collected, added to methanol (1:3) and vortexed to precipitate proteins. After recentrifugation at 12,000 rpm for 5 min, the supernatant was measured by using HPLC with a mobile phase of 0.1% trifluoroacetic acid/methanol (92:8, v/v) and a flow rate of 0.7 ml/min.

### β-Lactamase Activity Assay

β-lactamase activity was measured by using a β-lactamase assay kit (YIASEN Biotech Co., Ltd., Shanghai, China) according to the manufacturer’s instructions. Briefly, mid-log growth-phase bacteria were diluted to 1 × 10^8^ CFU/ml and treated with I1W or L12W at concentrations ranging from 3.13 to 50 μM for 12 h at 37°C. After induction, the cultures were harvested by centrifugation at 5,000 rpm at 4°C, washed three times with PBS buffer (pH 7.2), and then resuspended in the same buffer. The cells were sonicated on ice for 15 min, and the cell lysates containing β-lactamase were collected by centrifugation at 12,000 rpm for 30 min at 4°C. Fifty microliters of the prepared β-lactamase-containing lysates was mixed with 50 μl of assay buffer containing nitrocefin and reacted at room temperature in a dark environment for 20 min. Nitrocefin hydrolysis was measured spectrophotometrically at 490 nm. Concurrently, the total protein content was determined with a Bradford assay using the same supernatant. One milliunit of β-lactamase is defined as 1 nanomole of nitrocefin hydrolyzed per minute per microgram of protein.

### Statistical Analysis

All the experiments were performed in triplicate. The results are generally expressed as the means and standard errors. The paired Student’s *t*-test was used to test for significance. The significance is indicated as ^∗^ for *p* < 0.05 and ^∗∗^ for *p* < 0.01.

## Results

A previous study showed that Trp-containing peptides had antibacterial activity against the MRPA0108 strain, with MIC values ranging from 6.25 to 25 μM (Supplementary Table S1; Han et al., 2020). Among them, I1W and I4W exhibited higher antibacterial activity than the other peptides, especially L11W and L12W with fourfold higher MICs, and L5W, I1WL5W, and I4WL5W showed moderate antibacterial activity. As previously reported, neither of the peptides showed hemolytic activity at the tested concentrations of 500 μM (Supplementary Table S1; Bi et al., 2013).

### Effect of the Trp-Containing Peptides on QS

**FIGURE 1:**
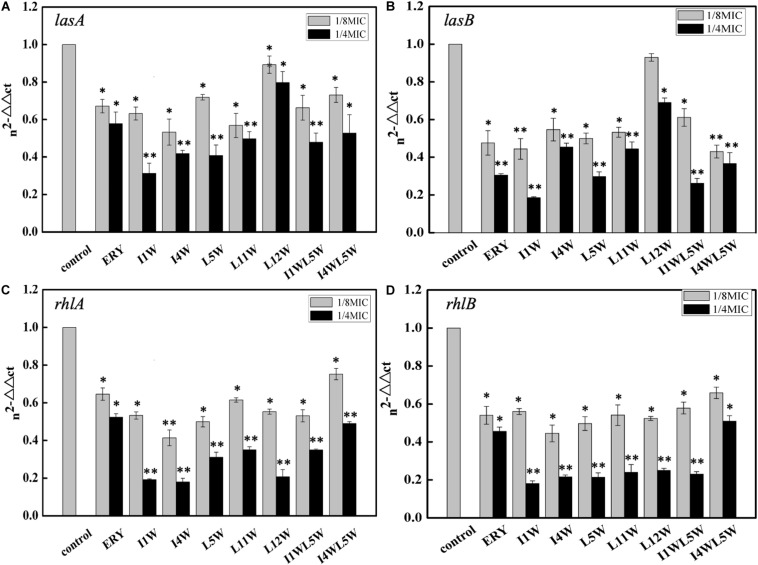
Relative expression of quorum-sensing genes measured by real-time qPCR. **(A)** *lasA*; **(B)** *lasB*; **(C)** *rhlA*; **(D)** *rhlB*. The MRPA0108 cells were treated in the absence (control) and presence of 1/4 × MIC and 1/8 × MIC of the Trp-containing peptides for 24 h. The fold changes of QS gene expression were normalized to the level of *rpsL* gene expression and then quantitated relative to gene expression of control was normalized to 1 using the comparative Ct method. Results represent the means and *SD* from triplicate experiments. *P < 0.05 and **P < 0.01 indicate statistically significant differences between peptide and control.

The relative expression of key virulence and QS genes were investigated in MRPA0108 after Trp-containing peptide treatment. The RT-qPCR results revealed that four virulence factor regulatory genes in the MRPA0108 QS system were significantly downregulated upon peptide exposure, namely, *lasA*, *lasB*, *rhlA*, and *rhlB*. In particular, the *rhlA* and *rhlB* relative expression levels were extremely low after peptide treatment (Figure 1). Trp-containing peptides at 1/8 × MIC inhibited the gene expression of *las*A 10.7–47.8% and *lasB* 7.1–55.6%. As the concentration of peptide increased to 1/4 × MIC, the expression of the *lasA* and *lasB* genes was downregulated by 20.3–68.8 and 31.0–81.5%, respectively (Figures 1A,B). Among the peptides, I1W exhibited the highest inhibitory effect. In the same way, the gene expression of *rhl*A and *rhl*B was downregulated 24.8–50.7 and 34.1–55.5%, respectively, in MRPA0108 cells upon Trp-containing peptide treatment at 1/8 × MIC and downregulated 51.1–82.1 and 49.1–82.0%, respectively, at a peptide concentration of 1/4 × MIC (Figures 1C,D).

**FIGURE 2:**
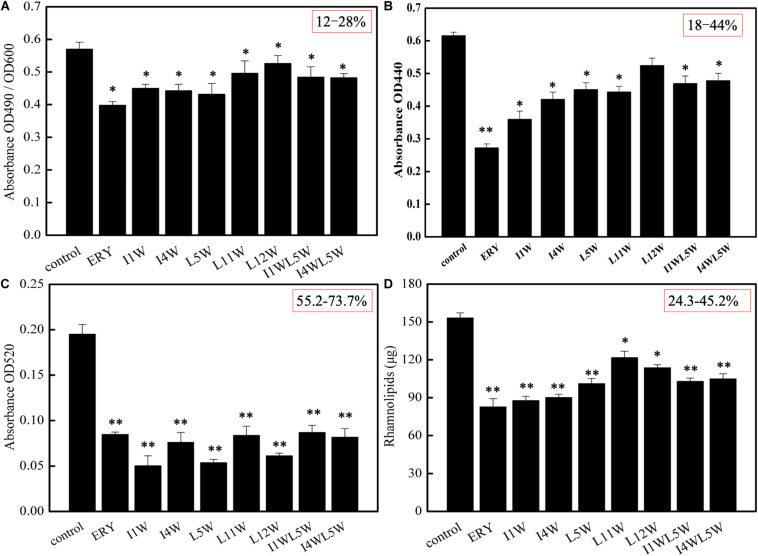
The production of virulence factors regulated by QS system in MRPA0108. **(A)** *lasB* elastase; **(B)** *lasA* protease; **(C)** pyocyanin; **(D)** rhamnolipid. The MRPA0108 cells were treated in the absence (control) and presence of 1/4 × MIC of the Trp-containing peptides for 24 h. The production of virulence factors was examined, respectively. Results represent the means and *SD* from triplicate experiments. *P < 0.05 and **P < 0.01 indicate statistically significant differences between peptide and control.

The effect of the Trp-containing peptides on the activity of elastase and alkaline protease, virulence factors regulated by the *las* QS system in MRPA0108, was examined by using the ECR method and azocasein method, respectively. The LasB elastase activity of MRPA0108 after exposure to the Trp-containing peptides is shown in Figure 2A. Compared to that of the control, the LasB elastase activity of MRPA0108 treated with I1W, I4W, and L5W at 1/4 × MIC decreased by 21.0, 22.3, and 24.1%, respectively, and decreased less than 20% with the other Trp-containing peptides. The activity of alkaline protease also decreased by 18–44% in MRPA0108 treated with Trp-containing peptides at 1/4 × MIC compared to that in the control (Figure 2B). These results suggested that the Trp-containing peptides reduced the production of the two virulence factors regulated by the *las* system and that the inhibitory effect increased as the peptide concentration increased.

Pyocyanin and rhamnolipid are important virulence factors regulated by the *rhl* QS system in MRPA0108. Pyocyanin induces neutrophil apoptosis and damages neutrophil-mediated host defense, resulting in severe toxic effects in humans (Fothergill et al., 2007). The results showed that the Trp-containing peptides significantly inhibited pyocyanin production in a dose-dependent manner. Pyocyanin production was reduced maximally to 73.7, 69.4, and 63.1% over that in the untreated control after the I1W, L5W, and L12W treatments at 1/4 × MIC, respectively. The other peptides produced approximately 55% inhibition of pyocyanin production (Figure 2C). Similar concentration-dependent results were observed with the positive control. Rhamnolipid production was examined by using the orcinol method. As shown in Figure 2D, I1W and I4W inhibited rhamnolipid production by 45.2 and 43.9%, respectively, and 40.7, 38.8, 37.5, and 24.3% inhibition was observed with L5W, I1WL5W, I4WL5W, and L11W, respectively. This result is consistent with the RT-qPCR results, suggesting that the inhibitory effect of the Trp-containing peptides on virulence factor production regulated by the *rhl* system is better than that on virulence factor production regulated by the *las* system.

### Effect of the Trp-Containing Peptides on MRPA 0108 Biofilms

#### The Trp-Containing Peptides Reduced MRPA 0108 Biofilm Formation

**FIGURE 3:**
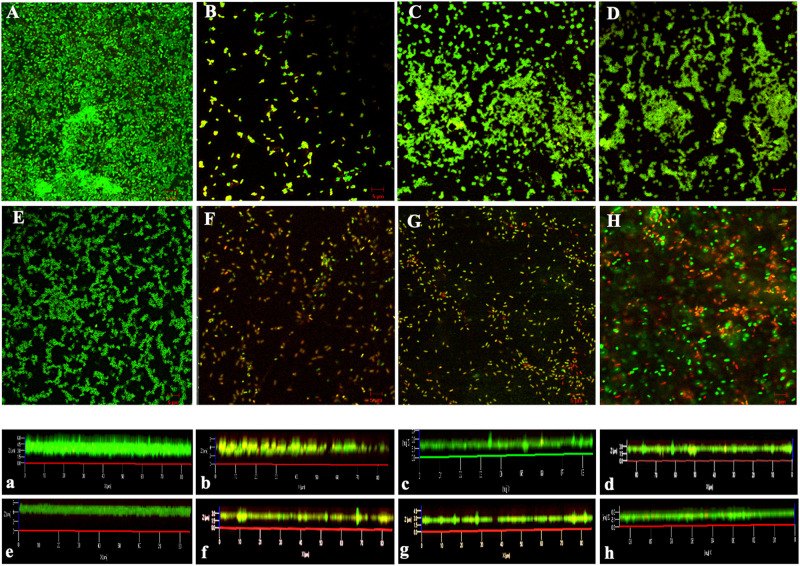
Confocal laser scanning microscopy images of MRPA0108 biofilms. The MRPA0108 cells were incubated at 37°C for 24 h in the absence (control) and presence of the Trp-containing peptides, respectively, and then stained using a LIVE/DEAD BacLight ^TM^ bacterial viability kit. Dead cells are stained red, whereas live cells are stained green. **(A)** Control; **(B)** erythromycin; **(C–E)** 1 × MIC of I1W, I4W, and L5W; **(F–H)** 2 × MIC of I1W, I4W, and L5W. **(a–h)** Illustrate an average thickness of biofilms. The micrographs are representative of two independent experiments.

QS controls biofilm formation in addition to modulating virulence and pathogenicity. Biofilms greatly enhance the microbial capacity to resist antimicrobial agents (Sergey et al., 2018). Confocal laser scanning microscopy was used to observe biofilms by using SYTO9 and PI, membrane permeability-sensitive DNA-binding dyes as markers. As shown in Figure 3, the biofilm of the MRPA0108 strain had a dense highly aggregated intercellular structure (Figure 3A), but it became sparse with the addition of I1W, I4W, or L5W (Figures 3C–H), suggesting that the peptides inhibited MRPA0108 biofilm formation in a concentration-dependent manner. The average thickness of the 1-day old MRPA0108 biofilm was 6 μm (Figure 3a), but an average biofilm thickness of 1.5–2.5 μm (Figures 3c–h) was observed after treatment with low concentrations of the Trp-containing peptides I1W, I4W, and L5W. The percentage of dead/live cells was 8.7% for control, 68.9% for erythromycin treatment, 29.9 and 47.9% for I1W at a concentration of 1 × MIC and 2 × MIC, 31.2 and 41.7% for I4W at a concentration of 1 × MIC and 2 × MIC, and 21.4 and 32.5% for L5W at a concentration of 1 × MIC and 2 × MIC, respectively.

**FIGURE 4:**
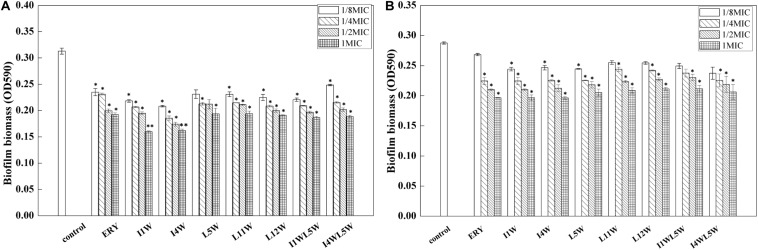
Effect of the Trp-containing peptides on the MRPA0108 biofilm. **(A)** Effect of the peptides on biofilm formation: the MRPA0108 cells were incubated at 37°C for 24 h in the absence (control) and presence of a series concentration of the peptides, respectively. Biofilm biomass was quantified by crystal violet dye (CV) staining. **(B)** Effect of the peptides on 24 h mature biofilm: Biofilms of MRPA 0108 cells were grown for 24 h in the absence of the peptide, and then planktonic bacteria were removed and the 24 h mature biofilm was treated with a series concentration of the peptides at 37°C for 24 h. Biofilm biomass was quantified as described above. Results represent the means and *SD* from triplicate experiments. *P < 0.05 and **P < 0.01 indicate statistically significant differences between peptide and control.

Next, the biofilm-forming capability of the MRPA0108 strain was observed after incubation with Trp-containing peptides at concentrations ranging from 1/8 × MIC to 1 × MIC for 24 h. The results showed that the peptides significantly decreased biofilm formation in a dose-dependent manner. A maximum reduction of 53% occurred with the treatment with I1W at 1 × MIC. I1W and I4W at a concentration of 1/8 × MIC inhibited 30 and 33% of biofilm formation, respectively. As peptide concentration increased onefold to eightfold (1/4 × MIC, 1/2 × MIC and 1 × MIC), inhibition of biofilm formation increased 33–53 and 40–48% compared to the control, respectively. Similar observations were recorded with the other peptides. Erythromycin, the positive control, exhibited inhibition similar to that by L5W, L11W and L12W (Figure 4A). For 1-day mature biofilms, the Trp-containing peptides at concentrations of 1 × MIC reduced the attached biofilm biomass by 26–31%, but the mature biofilm was only reduced by 11–15% when the peptide concentration decreased to 1/8 × MIC (Figure 4B), indicating that the Trp-containing peptides at a low concentration had no effect on the dispersal of the preformed biofilms compared with the control except for I1W and I4W.

#### The Trp-Containing Peptides Inhibited the Swarming Motility of MRPA0108 Biofilms

Swarming motility is vital in the initial attachment of bacterial cells to the matrix during biofilm formation. In the present study, the swarming motility of MRPA0108 cells was tested by measuring the diameter of colony growth in a semisolid medium. The MRPA0108 cells exhibited significantly reduced pilus-mediated swarming upon treatment with the Trp-containing peptides at 1/4 × MIC, with an inhibition of 90% for I1W, 85% for I4W, and 80% for other peptides compared to that of the control (Supplementary Figure S1). The positive control erythromycin also induced a significant reduction of 85% in swarming motility for MRPA0108 compared to that of the control.

#### The Trp-Containing Peptides Reduced the Polysaccharide Content of MRPA0108 Biofilms

**FIGURE 5:**
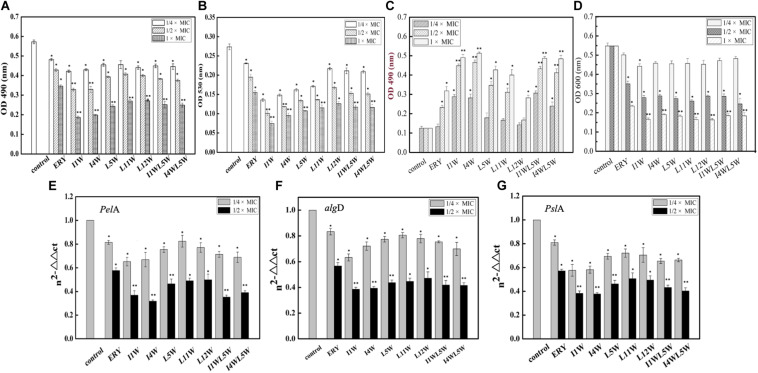
Effect of the Trp-containing peptides on polysaccharide biosynthesis of MRPA0108 biofilm: extracellular polysaccharide **(A)**, alg-**(B)**, and *Psl* polysaccharide **(C,D)** was assessed as described in the “Materials and Methods” section. **(E–G)** Expressions of biofilm polysaccharide-associated genes *Pel*A **(E)**, *alg*D **(F)**, and *Psl*A **(G)** was assessed by real-time qPCR. Fold changes of gene expression were normalized to the level of *rpsL* gene and quantitated relative to gene expression of control was normalized to 1 using the comparative Ct method. Results represent the means and *SD* from triplicate experiments. *P < 0.05 and **P < 0.01 indicate statistically significant differences between peptide and control.

Exopolysaccharide is an important component of biofilms to protect them from antimicrobial agents. It was hypothesized that the Trp-containing peptides at subinhibitory concentrations would decrease the forming of MRPA0108 biofilm. As shown in Figure 5A, 1/4 × MICs of I1W and I4W interfered with the exopolysaccharide production in MRPA0108 by 26.3 and 25.0%, respectively. As the peptide concentration increased onefold, the exopolysaccharide production was reduced by 42.6 and 42.4%, respectively, and reduced by 67.3 and 65.2% when the peptide concentration increased to 1 × MIC, respectively.

Exopolysaccharide includes at least three polysaccharides, such as alginate, Pel, and Psl, to attach to the surface of the planktonic cells of *P. aeruginosa* (Ryder et al., 2007; Ghafoor et al., 2011). Alginate is a polyuronide that enhances bacterial cell adhesion to the surface of the matrix. The results showed that the Trp-containing peptides significantly reduced alginate production in a concentration-dependent manner (Figure 5B). I1W and I4W at 1/4 × MIC inhibited alginate production by 53 and 47% of and inhibited alginate production by 74 and 68% when the peptide concentration increased to 1 × MIC. L5W, L11W, L12W, I1WL5W, and I4WL5W exhibited lower inhibition than I1W and I4W. Psl polysaccharide synthesis in MRPA0108 cells was determined by the CR binding assay (Ma et al., 2006). As more Psl containing either -1,3- or -1,4-glucopyranosyl units in the culture supernatant binds to the liquid Congo red, the A 490 nm value decreases, but the A600 nm value increases. As shown in Figures 5C,D, the A600 and A490 nm values were slightly decreased and increased, respectively, in MRPA0108 treated with the low concentration of the Trp-containing peptides (1/4 × MIC) compared to those in the control. When the peptide concentration increased to 1 × MIC, the A490 nm value increased threefold to fivefold compared to those in the control, and the A600 nm value decreased twofold to fourfold, suggesting that the Trp-containing peptides inhibited Psl synthesis in MRPA0108 cells in a concentration-dependent manner.

#### The Trp-Containing Peptides Downregulated the Expression of Polysaccharide Synthesis-Related Genes in MRPA0108 Biofilms

The gene expression of three biofilm polysaccharide-associated genes, *pslA*, *alginate* (*algD*), and *pelA*, was quantified using real-time qPCR. The results showed that the Trp-containing peptides significantly reduced the transcription level of the three genes in MRPA0108 cells in a concentration-dependent manner (Figures 5E–G). Expression of *pelA*, *algD* and *PslA* genes decreased by 28–41, 21–39, and 20–38% compared with the control levels, respectively, after MRPA0108 cells were incubated with the 1/4 × MIC Trp-containing peptides for 24 h. In addition, the gene expression levels of *pelA*, *algD* and *PslA* s were inhibited by 50–61, 53–62, and 52–65%, respectively, compared with the control levels when the peptide concentration increased to 1/2 × MIC. I1W and I4W exhibited higher inhibition effect than other peptides and the positive control erythromycin.

### The Trp-Containing Peptides Exhibit Synergistic Action in Combination With Antibiotics

#### The Trp-Containing Peptides Exhibited Synergy With Antibiotics

**FIGURE 6:**
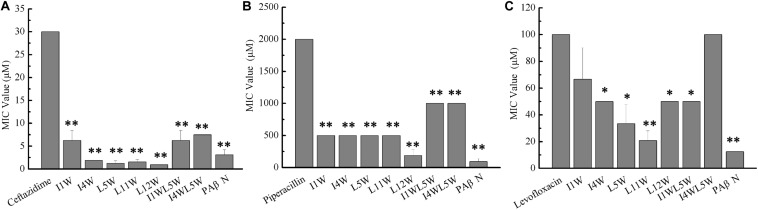
Antibacterial activity of antibiotics in combination with the Trp-containing peptides. Bacterial cultures were treated with a series of concentration of antibiotics ceftazidime **(A)**, piperacillin **(B)**, and levofloxacin **(C)** in the absence (control) and presence of a 1/4 × MIC peptide concentration at 37C overnight. The OD600 was recorded using a microtiter plate reader. The MIC was defined as the lowest antibiotics concentration that inhibited 95% of the bacterial growth. Phenylalanyl arginyl β-naphthylamide (PAβN), a broad-spectrum inhibitor for efflux pump, was used as a positive control. Each data point represents an average of six independent experiments. *P < 0.05 and **P < 0.01 indicate statistically significant differences between antibiotics alone and antibiotics in combination with peptide.

We investigated the synergistic effect of the Trp-containing peptides in combination with commercially available antibiotics, including ceftazidime, piperacillin and levofloxacin by using the checkerboard method. These antibiotics in combination with a low peptide concentration equivalent to 1/4 × MIC showed noticeably improved antimicrobial activity, with 8–32-fold reduced MIC values for ceftazidime and 2–11-fold reduced MIC values for piperacillin, but the antimicrobial activity of levofloxacin exhibted no significant improvement, with only 0–4-fold reduced MIC values (Figure 6). Ceftazidime and piperacillin showed better antibacterial activity against the MRPA0108 strain in combination with 1/4 × MIC L12W, whose MIC values decreased from 30 to 0.94 μM and from 500 to 187 μM, respectively. The FICI defining synergy between the antibacterial agents is shown in Table 1. According to the previous studys (Hollander et al., 1998; Yoon et al., 2004), FICI ≦ 0.5 were defined as synergy, and 0.5 < FICI ≦ 1.0 and 1.0 < FICI ≦ 2.0 were defined as addition and indifference, respectively. The antibacterial activities of ceftazidime were synergistic with all of the tested Trp-containing peptides, with FICI values of 0.23–0.43, and L5W, L11W, and L12W exhibited the highest synergistic effects. Piperacillin had a synergistic effect with I1W, I4W, L5W, L11W, and L12W, with FICI values of 0.28–0.49, but additive action with I1WL5W and I4W5W, with FICI values of 0.59 and 0.68, respectively. Levofloxacin exhibited only additive action with the tested Trp-containing peptides, with FICI values of 0.51–0.87, except I4W5W, with which levofloxacin had an indifferent effect (FICI value: 1.12). Notably, although L11W and L12W alone had the lowest antibacterial activity against the MRPA0108 strain because of their low membrane disruption ability, they showed the highest synergy with ceftazidime and piperacillin.

**TABLE 1:** The fractional inhibitory concentration index (FICI) of the Trp-containing peptides in combination with antibiotics against MRPA0108.

Antibiotics	FICI						
	I1W	I4W	L5W	L11W	L12W	I1WL5W	I4WL5W
Ceftazidime	0.43	0.31	0.23	0.25	0.23	0.37	0.43
Piperacillin	0.49	0.49	0.37	0.34	0.28	0.59	0.68
Levofloxacin	0.87	0.75	0.62	0.5	0.75	0.75	1.12

*Synergy is defined as FICI ≤ 0.5, 0.5 < FICI ≤ 1.0 as additive, 1.0 < FICI ≤ 4.0 as indifferent, and FICI > 4.0 as antagonism. Experiments were performed in triplicate (n = 6). Means ± the SD are shown; p ≤ 0.001.*

#### The Trp-Containing Peptides Increased the Antibacterial Activity of Antibiotics by Inhibiting β-Lactamase Activity in MRPA0108 Cells

**FIGURE 7:**
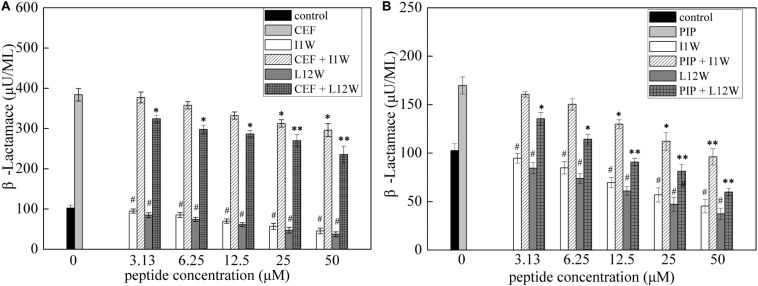
β-lactamase activity in the MRPA0108 cells treated with the Trp-containing peptides in combination with β-lactama antibiotics ceftazidime **(A)** and piperacillin **(B)**. Bacteria were treated with a series of concentration of I1W and L12W for 12 h at 37°C. β-lactamase activity in the prepared β-lactamase-containing lysates was measured by using a β-lactamase assay kit. *P < 0.05 and **P < 0.01 indicate statistically significant differences between antibiotics alone and antibiotics in combination with peptide, and ^#^*P* < 0.01 between peptide alone and antibiotics alone, respectively. CEF, ceftazidime; PIP, piperacillin.

Both ceftazidime and piperacillin are β-lactam antibiotics. To understand the synergistic mechanism of I1W and L12W in combination with ceftazidime and piperacillin, β-lactamase activity in MRPA0108 cells was detected. The results showed that the β-lactamase activity increased fourfold and 1.8-fold in MRPA0108 cells treated with ceftazidime and piperacillin alone compared to that in the control, respectively (Figure 7). I1W and L12W alone at concentrations greater than 12.5 μM reduced β-lactamase activity compared to that in the control, but at low concentrations, they had no effect on β-lactamase activity. The combination of I1W and L12W with ceftazidime or piperacillin decreased β-lactamase activity in a concentration-dependent manner compared to that with the antibiotics alone. Specifically, the combination of ceftazidime or piperacillin with L12W even at low concentrations (3.13 μM), significantly decreased β-lactamase activity by 15.8 and 23.5%, respectively. L12W at 25 μM combined with ceftazidime and piperacillin caused a reduction of 31.6 and 58.8% in β-lactamase activity, respectively. These results suggested that the ceftazidime/piperacillin-peptide combination displayed highly synergistic activity on β-lactamase production.

#### The Trp-Containing Peptides Inhibit the Gene Expression of Efflux Pumps, Resulting in an Increase in Antibiotics in MRPA0108 Cells

**FIGURE 8:**
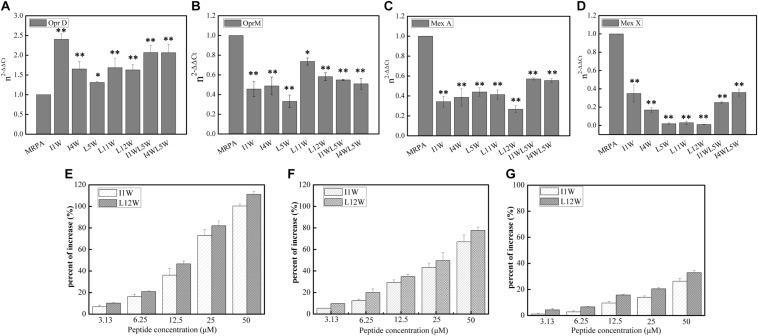
Effect of the Trp-containing peptides on the antibiotics content in MRPA0108 cells. **(A–D)** Relative expression of porin gene *OprD* **(A)**, efflux pump gene *OprM* **(B)**, *MexA* **(C)**, and *MexX* **(D)** was measured by real-time qPCR. Fold changes of gene expression were normalized to the level of *rpsL* gene and quantitated relative to gene expression of control was normalized to 1 using the comparative Ct method. **(E–G)** The content of antibiotics ceftazidime **(E)**, piperacillin **(F)**, and levofloxacin **(G)** in MRPA0108 cells treated with the Trp-containing peptides was measured by HPLC. Results represent the average of at least four independent experiments *SD* (*P < 0.05 and **P < 0.01).

Previous studies have clearly shown that QS systems are involved in antimicrobial resistance regulation via efflux pump genes, including MexAB-OprM and MexXY/OprM (Evans et al., 1998; Minagawa et al., 2012; Rasamiravaka and Jaziri, 2016). Many of the clinical strains of *P. aeruginosa* with high expression of the *lasI* and *lasR* genes exhibit elevated *MexA/B* gene expression, resulting in high resistance to currently used broad-spectrum drugs (Pourmand et al., 2013). In our study, relative expression of the *MexA*, *MexX*, and *OprM/D* genes was detected using RT-qPCR. The results showed that the transcription level of the *OprD* gene significantly increased and that the expression of the *OprM*, *MexX*, and *MexA* genes was reduced upon Trp-containing peptide addition at 25 μM in MRPA0108 cells (Figures 8A–D). The peptides increased the gene expression of *OprD* 1.3–2.5-fold compared to that in the control and decreased the expression of the *OprM*, *MexX* and *MexA* genes by 21–68, 40–75, and 70–90%, respectively. However, I1W at its MIC (6.25 μM) reduced the *OprD/M* gene expression by 27 and 22.3%, respectively, and L12W at its MIC (25 μM) reduced the *OprD/M* gene expression by 72 and 41.3%, respectively (Supplementary Figure S2). The results suggested that the Trp-containing peptides were able to increase antibiotic content in cells by inhibiting the expression of efflux pump genes.

Subsequently, we detected the contents of the antibiotic ceftazidime, piperacillin, and levofloxacin in MRPA0108 cells treated with or without the Trp-containing peptides I1W and L12W by RP-HPLC. As shown in Figures 8E–G, I1W and L12W increased the three antibiotics contents inside the MRPA0108 cells in a concentration-dependent manner. I1W caused an increase of 7.5-100% for ceftazidime, 8–80% for piperacillin and 0.01–22% for levofloxacin. L12W caused an increase of 8–100% for ceftazidime, 5–62% for piperacillin and 2.1–38% for levofloxacin. Similarly, I1W at its MIC increased the content of ceftazidime by 19%, piperacillin by 17% and levofloxacin by 1% in MRPA0108 cells, but L12W at its MIC increased the content of ceftazidime by 81%, piperacillin by 58%, and levofloxacin by 23%. These results indicated that the Trp-containing peptides reduced antibiotic efflux from MRPA0108 cells by inhibiting efflux pump gene expression, thus increasing the antibacterial activity of these antibiotics against the MRPA0108 strain.

## Discussion

QS systems in *P. aeruginosa* play a role in the production of virulence factors, bacterial swarming motility and development of biofilm and are a potential target as a new therapeutic approach in bacterial infection and biofilm formation (Rasamiravaka and Jaziri, 2016). The present study focused on the QS attenuation potential of Trp-containing peptides and the role of the peptides on the virulence and biofilm formation regulated by quorum sensing of MRPA. In *P. aeruginosa*, QS activates virulence factors proteases and elastase regulated by the *las* system, and pyocyanin and rhamnolipids regulated by the *rhl* system (Pattnaik et al., 2018). This study confirms that Trp-containing peptides affect global regulatory QS signaling in MRPA0108. In this study, four virulence factor regulatory genes in the QS system of MRPA0108 were significantly downregulated in a concentration-dependent manner upon Trp-containing peptide exposure, namely, *lasA*, *lasB*, *rhlA*, and *rhlB*. The peptides at low concentrations (1/4 × MIC or 1/8 × MIC) strongly decreased the relative expression levels of the *rhlA* and *rhlB* genes. Consistent with these results, the Trp-containing peptides significantly inhibited the production of the LasB elastase and LasA protease regulated by the *las* system, with the highest inhibition being 24 and 44% at peptide concentrations of 1/4 × MIC, respectively. The production of pyocyanin and rhamnolipids regulated by the *rhl* system was reduced maximally to 73 and 44% over that of the untreated control after the peptide treatments at 1/4 × MIC, respectively. Compared with the effect on elastase and total protease production, the Trp-containing peptides showed more significant inhibitory effect on the production of pyocyanin and rhamnolipid, suggesting that the inhibitory effect of the peptides on the *rhl* system is better than that on the *las* system.

Davies et al. (1998) reported that the *lasI* mutant produced a flat and densely packed multilayer biofilm that was different from the large-aggregate wild-type biofilms and was sensitive to detergent treatment. Subsequent studies revealed that QS plays an important role in biofilm formation in *P. aeruginosa*, in which the *lasI/lasR* and *rhlR/rhlI* systems are expressed during the initial stage of biofilm formation and the maturation stage of *P. aeruginosa* biofilm development (Bjarnsholt et al., 2010; Wrafy et al., 2016). Among virulence factors produced by *P. aeruginosa*, pyocyanin, and rhamnolipids regulated by the *rhl* system are responsible for the deposition of extracellular DNA (eDNA), which is a major biofilm component of the EPS, microcolony formation and structural development in the early and late biofilm development stages, respectively (Pamp and Nielsen, 2007; Brackman and Coenye, 2015; Qadri et al., 2016; Silva et al., 2017). Thus, they are key mediators of bacterial persistence in *P. aeruginosa*. Rhamnolipid-deficient *P. aeruginosa* cannot maintain open spaces to aid nutrient diffusion through densely populated mature biofilms (Davey et al., 2003). The ability of the Trp-containing peptides to modify QS signaling and reduce the production of pyocyanin, proteases and rhamnolipids in MRPA0108 may influence critical downstream functions, such as biofilm formation. Confocal laser scanning microscopy and CV dye assays demonstrated that peptide-treated biofilms had a concentration-dependent decrease in biomass. A maximum reduction of 53% occurred with the treatment with I1W at 1 × MIC, and an average biofilm thickness of 6 μm decreased to 1.5 μm after treatment with a low concentration of I1W. For 1 day mature biofilms, Trp-containing peptides at concentrations ranging from 1/8 × MIC to 1 × MIC reduced the existing-biofilm biomass, but they were less effective in the degradation of 1-day mature biofilms and reduced 1-day-old MRPA0108 strain biofilms by only 15–30%. Although QS regulation of biofilms is complex and involves numerous intrinsic and environmental factors, such as the matrix density, EPS composition, cell number, and oxygen availability, rhamnolipids, proteases, and pyocyanin participate in optimal QS signaling of biofilm (Furiga et al., 2016).

At least three polysaccharides, Psl, Pel, and alginate are produced to stabilize the biofilm structure in *P. aeruginosa* (Ryder et al., 2007; Ghafoor et al., 2011). Our data also showed that the synthesis of alginate and Psl was significantly inhibited in MRPA0108 cells treated with the Trp-containing peptides for 24 h, and the transcription levels of the *pelA*, *algD*, and *pslA* genes were significantly downregulated. As a result, alginate and Psl production showed a significant reduction, with maximum inhibition of 53–74% and 12–74%, after treatment with Trp-containing peptides at 1 × MIC, respectively. Psl, a major biofilm matrix polysaccharide, can act as a signal to stimulate its own production and that of other biofilm components (alginate and Pel) via the intracellular secondary messenger molecule c-di-GMP, which is regulated by two diguanylate cyclases (Irie et al., 2012). On the other hand, Gilbert et al. found that the QS regulator *LasR* can regulate *psl* expression by binding itself to the promoter region of the psl operon (Gilbert et al., 2009). Here, peptide-driven downregulation of polysaccharide biosynthesis plays an important role in biofilm information, in which the Trp-containing peptides reduce the amounts of EPSs to decrease the biofilm structural stability and interfere with MRPA0108 biofilm formation.

The bacterial swarming motility assay further supported a significant effect of the peptides on bacterial flagellum-and pilus-mediated chemotaxis. QS regulates swarming motility in *P. aeruginosa* to affect both biofilm formation and established biofilm disruption (Overhage et al., 2007). Motility plays an important role in colonization and exploitation of new niches of *P. aeruginosa*. Swarming motility is a complex behavior influenced by a large number of different genes and has been linked to increased antibiotic resistance (Overhage et al., 2008). Rhamnolipids can modulate the intricate *P. aeruginosa* swarming motility patterns (Caiazza et al., 2005). Therefore, it was perhaps unsurprising that Trp-containing peptides affected the MRPA0108 swarming motility. The study of Overhage et al. (2007) demonstrated that a series of “swarming-negative” *P. aeruginosa* mutants obtained via the insertion of mini-Tn5 exhibited impaired biofilm formation, indicating the link between the two phenotypes.

Previous studies have demonstrated that combination of antibiotics with some cationic AMPs have synergistic effects (Maisetta et al., 2009; Kajal et al., 2015; Regmi et al., 2017). Such synergy increases the antibacterial activity of antibiotic and peptide and helps prevent bacterial resistance. Most cationic AMPs, such as magainin II, ranalexin, cecropin P1, indolicidin, and buforin II, adopt an amphiphilic α-helical confirmation and show membrane-disrupting activity (Giacometti et al., 2000; He et al., 2015). The increase of membrane permeability leads to easy cellular uptake of small molecule antibiotics and exerts synergistic effects. Ranalexin, a 20-residue AMP from the skin secretion of *Rana catesbiana Shaw*, was demonstrated to act in a synergistic manner with clarithromycin, polymyxin E, and doxycycline against *Staphylococcus aureus* because of its membrane-disrupting activity, leading to entry of hydrophobic compounds into the cell (Viljanen et al., 1991; Zhou and Peng, 2013). Similarly, magainin II was observed to be synergistic with the clinically used antibiotics amoxicillin, ceftriaxone, ceftazidime, piperacillin, and meropenem, but buforin II, cecropin P1 and indolicidin did not show synergistic effects in combinations with these antibiotics against *S. aureus* (Falla and Hancock, 1997; Zhou and Peng, 2013). Our previous study showed that the combination of penicillin, ampicillin and erythromycin with the Trp-containing peptides L11W, L12W, I1WL5W, and I4WL5W had a distinct synergistic effect against MDR *Staphylococcus epidermidis* (MRSE) *in vitro* and *in vivo*, but ceftazidime in combination with the peptides exhibited additive activity (Shang et al., 2019). Interestingly, the present study demonstrated that the combination of all of the tested Trp-containing peptides with ceftazidime provided a synergistic effect against MRPA0108, with FICI values of 0.23–0.43. Piperacillin had a synergistic effect with I1W, I4W, L5W, L11W, and L12W, with FICI values of 0.28–0.49, but additive action with I1WL5W and I4W5W, with FICI values of 0.59 and 0.68, respectively. Levofloxacin exhibited only additive action with the tested Trp-containing peptides, with FICI values of 0.51–0.87, except I4W5W, which had an indifferent effect (FICI value: 1.12). In combination with a low peptide concentration equivalent to 1/4 × MIC, ceftazidime and piperacillin showed improved antimicrobial activity, with 32-fold- and 11-fold-reduced MIC values for ceftazidime and piperacillin, respectively, but only fourfold reduced MIC values for levofloxacin. Notably, although L11W and L12W alone had the lowest antibacterial activities against the MRPA0108 strain because of their low membrane disruption ability, they showed the highest synergy with ceftazidime and piperacillin. In addition, Reffuveille et al. (2014) demonstrated that the combinations of a broad-spectrum antibiofilm peptide at a subinhibitory levels and the antibiotics ceftazidime and ciprofloxacin prevented biofilm formation in *P. aeruginosa* and triggered cell death in mature *P. aeruginosa* biofilms, indicating a novel strategy to potentiate antibiotic activity against biofilms formed by multidrug-resistant pathogens.

In *Pseudomonas*, β-lactamases production is the most important mechanism of antibiotic resistance to penicillin, monobactams, and cephalosporins (Berrazeg et al., 2015; Bush and Bradford, 2016). At present, in addition to developing novel antibiotics by modifying the structure of β-lactams, the coadministration of a β-lactam antibiotic together with other compounds or biomolecules to inhibit β-lactamase is another relevant strategy (Peppoloni et al., 2020). Our results showed that β-lactamase activity increased fourfold and 1.8-fold in MRPA 0108 cells treated with ceftazidime or piperacillin alone, respectively, but the tested Trp-containing peptides I1W and L12W in combination with ceftazidime or piperacillin significantly decreased β-lactamase activity. Specifically, the β-lactamase activity was reduced by 31.6 and 58.8% in MRPA0108 treated with L12W along with ceftazidime or piperacillin, respectively. These results suggested that the ceftazidime/piperacillin-peptide combination displayed highly synergistic activity on β-lactamase production. Both the ceftazidime and piperacillin used in the present study are β-lactam antibiotics. In *P. aeruginosa*, *AmpC* encoding β-lactamase is regulated by *AmpR*, the *AmpC* β-lactamase regulator. The previous studys found that *AmpR* altered the production of β-lactamase by upregulating *AmpC* gene expression. *AmpR*, the LysR-type transcriptional regulator, not only regulates *AmpC* transcription by changing its conformation to modulate RNA polymerase activity (Ito et al., 2018; Torrens et al., 2019), but also controls the expression of 313 genes including metabolic, antibiotic-resistance and virulence genes (Kong et al., 2005). *AmpR* plays an important role in multiple virulence mechanisms by positively regulating the gene expression of *lasB* and *rhlR* and downstream genes (Lister et al., 2009; Balasubramanian et al., 2012). Moreover, mutation of *AmpR* results in a high expression of β-lactamases and higher levels of the virulence factors extracellular proteases and pyocyanin through quorum sensing (Kong et al., 2005). However, little is known about how QS signal molecules impact chromosomal AmpC β-lactamases. Our present results suggest a positive correlation between β-lactamase production inhibition and *las/rhl* system downregulation by the tested Trp-containing peptides. However, whether inhibition of β-lactamase production by the peptides results from its regulation of the QS system remains to be fully elucidated. Notably, further studies about the effect of the peptides on β-lactamases expression modulated by *AmpC*/*AmpR* will be helpful for further understanding the complex molecular mechanisms.

In addition to the production of β-lactamase, the expression of antibiotic multidrug efflux pumps, such as the MexAB/OprM, MexCD/OprJ, MexEF/OprN, and MexXY/OprM systems, intrinsically high levels of antibiotic resistance occur through the extrusion of a broad range of substrates, including antibiotics and chemotherapeutic agents in *P. aeruginosa* (Pourmand et al., 2013; Rasamiravaka and Jaziri, 2016). MexB, MexD, MexF, and MexY, which are located in the inner membrane, act in conjunction with the outer membrane proteins OprM, OprJ, and OprN through the periplasmic proteins MexA, MexC, MexE, and MexX, respectively. This study showed that the Trp-containing peptides I1W and L12W significantly inhibited the relative expression of the *OprM*, *MexX* and *MexA* genes but increased the level of transcription of the *OprD* gene. I1W at its MIC (6.25 μM) reduced *OprD/M* expression by 27 and 22.3%, and L12W at its MIC (25 μM) reduced *OprD/M* expression by 72 and 41.3%, respectively, suggesting that L12W had a better effect than I1W on inhibiting molecule efflux. Our results also confirm that the Trp-containing peptides I1W and L12W increased the contents of the three antibiotics inside the MRPA0108 cells by RP-HPLC. I1W at its MIC increased the content of ceftazidime by 19%, piperacillin by 17%, and levofloxacin by 1% in MRPA0108 cells, but L12W at its MIC increased the content of ceftazidime by 81%, piperacillin by 58%, and levofloxacin by 23%. These results indicated that the Trp-containing peptides reduced antibiotic efflux from MRPA0108 cells by inhibiting efflux pump gene expression, thus increasing the antibacterial activity of these antibiotics against MRPA0108. The biochemical targets of ceftazidime, piperacillin and levofloxacin are located intracellularly, and the mechanism of inhibiting β-lactamase activity and antibiotic multidrug efflux pump expression could have assisted the antibiotics in inhibiting their molecular targets.

Previous studies have clearly shown that the efflux pump system is associated with QS systems in *P. aeruginosa* (Evans et al., 1998; Minagawa et al., 2012). Many of the clinical MRPA strains overexpressing *lasI* and *lasR* exhibit elevated *MexAB* gene expression, resulting in high levels of resistance to currently used broad-spectrum antibiotics (Jeannot et al., 2008; Stickland et al., 2010). Similarly, a positive correlation between *mexXY/oprM* expression with the *las* system has also been found in some clinical MRPA strains (Pourmand et al., 2013). Furthermore, the exogenous addition of the signaling autoinducer C4-HSL enhanced *mexAB/oprM* operon expression in *P. aeruginosa*, indicating that C4-HSL accumulation in the medium directly induced *mexAB-oprM* operon expression in the stationary phase (Maseda et al., 2004). However, Evans et al. reported that the overexpressing *mexAB/oprM* in *P. aeruginosa* mutant strains lowered the expression of *lasI*, resulting in the decrease in the production of C12-HSL and virulence factors pyocyanin, elastase, and casein protease compared to wild-type strains (Evans et al., 1998; Rico et al., 2018). In agreement with the findings of Pourmand et al. (2013), the Trp-containing peptides inhibited *MexAB/OprM* expression and decreased virulence factor production regulated by *las* and *rhl*, indicating a positive correlation between *mexAB/oprM* expression and the *las* and *rhl* systems in the clinical strain MRPA 0108, in which decreased *las* and *rhl* gene expression result in reduced *mexA, mexB*, and *oprM* gene expression. This phenomenon may not be related simply to QS inhibition observed here with the peptides in *P. aeruginosa*, and the underlying reasons for the correlation between QS and the efflux pump *mexAB/oprM* remain to be elucidated.

## Conclusion

Assessing the effects of Trp-containing AMPs on virulence factors and biofilm formation and development regulated by QS in MRPA is essential for the development of potential drug targets. In the present study, the Trp-containing peptides exhibited significant QS attenuation potential in downregulating QS-regulated virulence factor production in MRPA. In addition, the Trp-containing peptides showed significant antibiofilm potential, suggesting their role in altering bacterial tolerance to conventional antibiotics. The present study will provide a new perspective for developing novel and highly effective antiinfective drugs targeting bacterial virulence.

## Data Availability Statement

The original contributions presented in the study are included in the article/Supplementary Material, further inquiries can be directed to the corresponding author/s.

## Ethics Statement

This study uses strains obtained from clinical specimens submitted to The First Affiliated Hospital of Dalian Medical University. The ethics committee of The First Affiliated Hospital of Dalian Medical University did not require the study to be reviewed or approved by an ethics committee because the samples were primarily isolated for clinical diagnosis, rather than for scientific research.

## Author Contributions

DS conceived and designed the experiments and wrote the manuscript. XH, WD, and ZK performed the experiments. FJ performed the isolation, identification, and antimicrobial susceptibility testing (AST) of MRPA0108. All authors contributed to the article and approved the submitted version.

## Conflict of Interest

The authors declare that the research was conducted in the absence of any commercial or financial relationships that could be construed as a potential conflict of interest.
