# Supplementary figures and images for: Trp-Containing Antibacterial Peptides Impair Quorum Sensing and Biofilm Development in Multidrug-Resistant Pseudomonas aeruginosa and Exhibit Synergistic Effects With Antibiotics
Source: Front Microbiol. 2021 Feb 11;12:611009. doi: 10.3389/fmicb.2021.611009 (PMC7906020; doi:10.3389/fmicb.2021.611009)

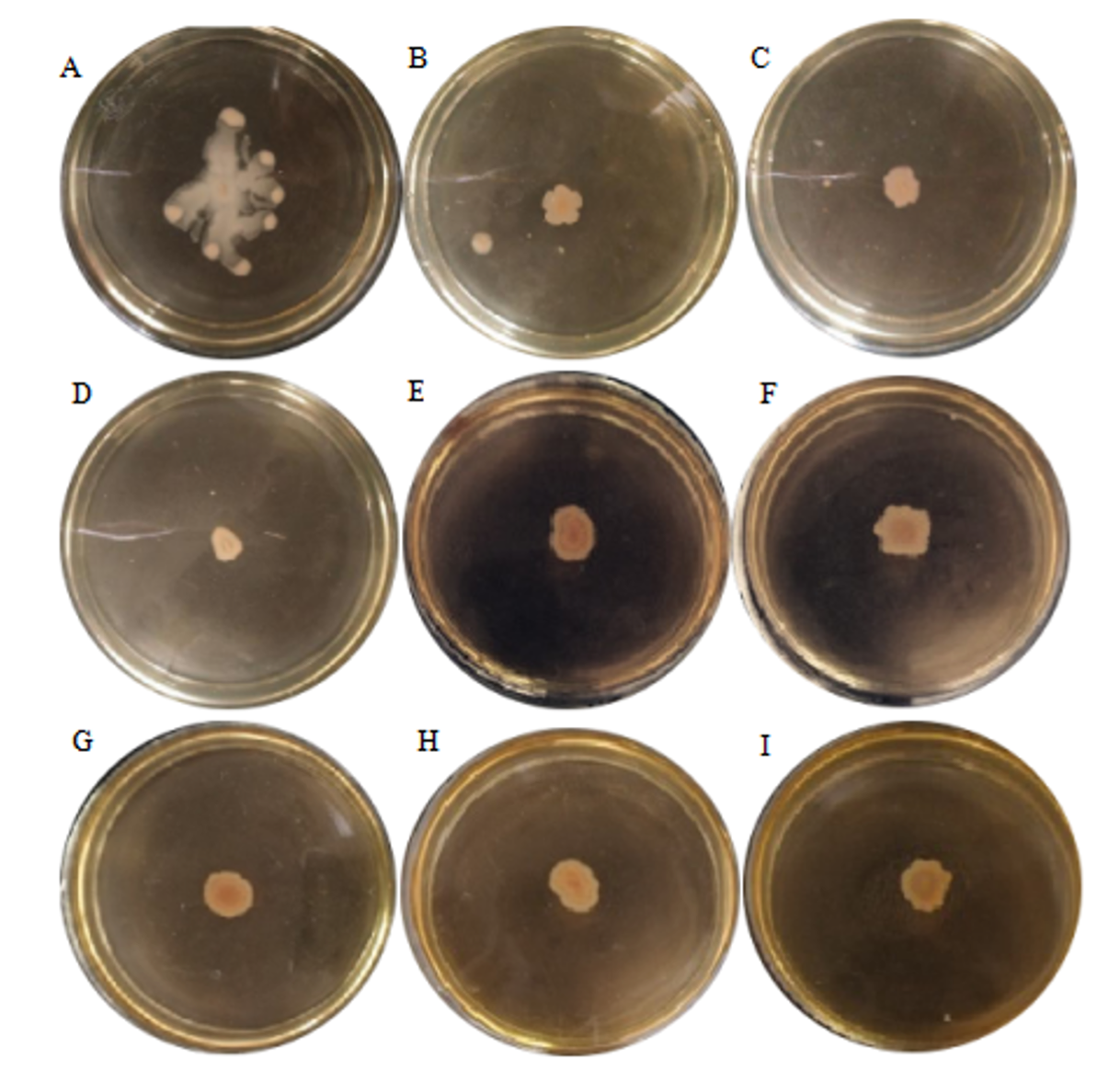

Supplement: Supplementary file 1 [file Image_1.TIFF]

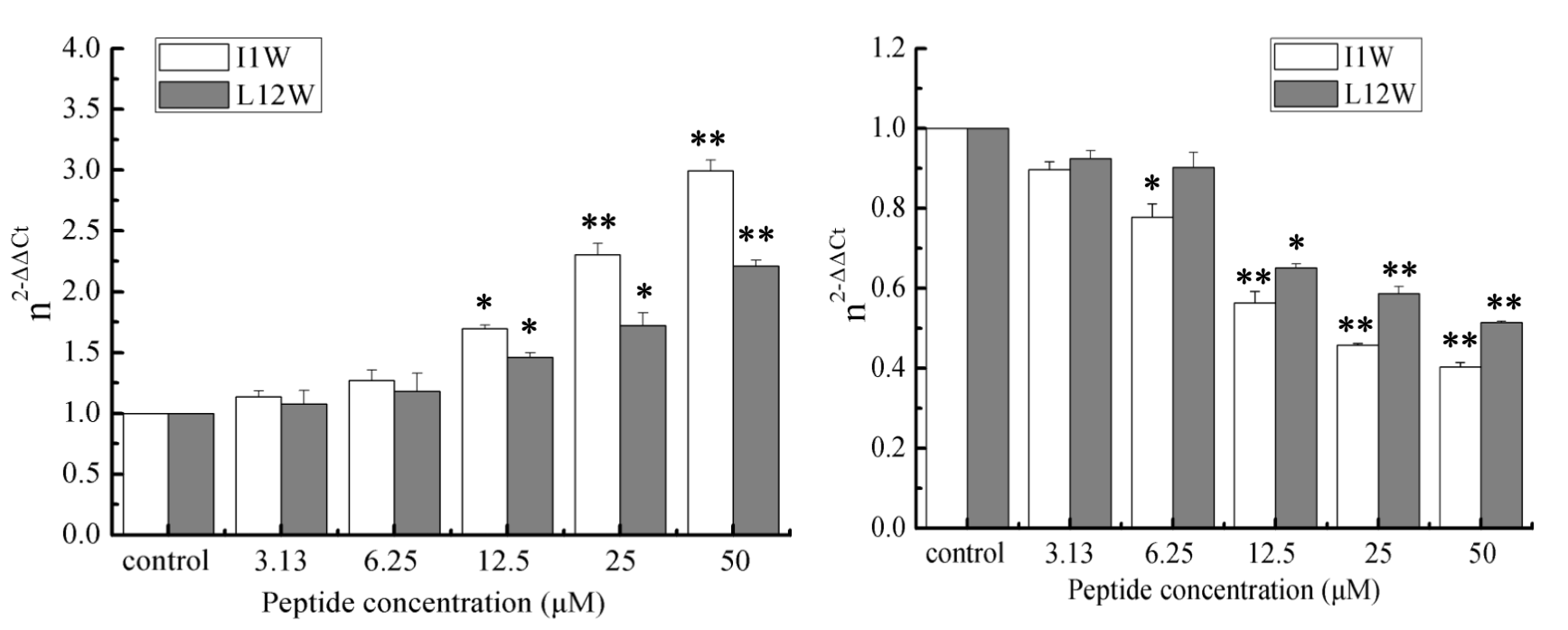

Supplement: Supplementary file 2 [file Image_2.TIFF]
