# Supplementary material for: Trp-Containing Antibacterial Peptides Impair Quorum Sensing and Biofilm Development in Multidrug-Resistant Pseudomonas aeruginosa and Exhibit Synergistic Effects With Antibiotics
Source: Front Microbiol. 2021 Feb 11;12:611009. doi: 10.3389/fmicb.2021.611009 (PMC7906020; doi:10.3389/fmicb.2021.611009)
Supplement: Supplementary file 3 [file Table_1.docx]

**Table S1** Biological characteristics and antibacterial activities of the Trp-containing antimicrobial peptides (32)

| peptide | Amino acid sequence | Amphipathicity | Hydrophobicity | Molecular mass (Da) | MIC(μM) | Hemolysis (μM) |
| --- | --- | --- | --- | --- | --- | --- |
| I1W | WKKILSKIKKLLK-NH_2_ | 0.82 | 12.57 | 1626.1 | 6.25 | > 500 |
| I4W | IKKWLSKIKKLLK-NH_2_ | 0.83 | 12.57 | 1626.1 | 6.25 | > 500 |
| L5W | IKKIWSKIKKLLK-NH_2_ | 0.83 | 13.33 | 1626.1 | 12.5 | > 500 |
| L11W | IKKILSKIKKWLK-NH_2_ | 0.82 | 13.33 | 1626.1 | 25 | > 500 |
| L12W | IKKILSKIKKLWK-NH_2_ | 0.83 | 13.33 | 1626.1 | 25 | > 500 |
| I1WL5W | WKKIWSKIKKLLK-NH_2_ | 0.83 | 13.15 | 1698.2 | 12.5 | > 500 |
| I4WL5W | IKKWWSKIKKLLK-NH_2_ | 0.82 | 13.15 | 1698.2 | 12.5 | > 500 |

The hydrophobicity of the peptide calculated using the hydrophobicity scale (Mant et al. 2009) was the total hydrophobicity (sum of the hydrophobicity indices for all residues) divided by the number of residues. Amphipathicity was determined by calculation of the hydrophobic moment (Eisenberg et al. 1982; Carver et al. 2003).
