# Supplementary material for: Trp-Containing Antibacterial Peptides Impair Quorum Sensing and Biofilm Development in Multidrug-Resistant Pseudomonas aeruginosa and Exhibit Synergistic Effects With Antibiotics
Source: Front Microbiol. 2021 Feb 11;12:611009. doi: 10.3389/fmicb.2021.611009 (PMC7906020; doi:10.3389/fmicb.2021.611009)
Supplement: Supplementary file 4 [file Table_2.docx]

Table S2 Primer sequences

| primer | Sequence (5’ to 3’) | Accession number |
| --- | --- | --- |
| *rps*L-F | CTGCAAGAGCACAGCGTAGT | NP_252958.1 |
| *rps*L-R | ACCCTGCTTACGGTCTTTGA |  |
| *las*A-F | CCGCTGAATGACGACCTGTTCC | NP_250562.1 |
| *las*A-R | CAGTGCTCCAGGTATTCGCTCTTG |  |
| *las*B-F | CGGCGGCAACCAGAAGATCG | NP_252413.1 |
| *las*B-R | CTGTCGTCGGTGCTGCTGTTC |  |
| *rhl*A-F | CTCGGTGGTGATGGCATTCG | NP_252169.1 |
| *rhl*A-R | GGTCTCGTTGAGCAGATGGC |  |
| *rhl*B-F | GAATTGTCACAACCGCACAGTATC | NP_252168.1 |
| *rhl*B-R | GCCTTCAGCCATCGAGCATC |  |
| *psl*A-F | AAACGCTACGGCTACAACAACC | NP_250921.1 |
| *psl*A-R | TATTCGCTGACCGCCTCCT |  |
| *pel*A-F | ACGCCCTTCGCCTATCTGT | NP_251754.1 |
| *pel*A-R | GAGGTCCATTACCTGGCTGTTC |  |
| *alg*D-F | CTCATCACCAGCCACGACA | NP_252230.1 |
| *alg*D-R | AGCACCAGCACATCGGAAC |  |
| *oprD*-F | CGACATCAGCAACACCACTT | NP_249649.1 |
| *oprD*-R | GCCGAAGCCGATATAATCAA |  |
| *oprM*-F | GTTCGGGTTCCTGGTTGTTC | NP_249118.1 |
| *oprM*-R | CGAGCAGGGTCAGGTAGTTGT |  |
| *mexA*-F | ACCTACGAGGCCGACTACCA | NP_249116.1 |
| *mexA*-R | GCGTACTGCTGCTTGCTCAC |  |
| *mexX*-F | ACCGTGCTGTTCCAGATCG | NP_250708.1 |
| *mexX*-R | CCTTGATCAGGTCGGCGTA |  |
